# Self-Catalyzed Growth of Vertical GaSb Nanowires on InAs Stems by Metal-Organic Chemical Vapor Deposition

**DOI:** 10.1186/s11671-017-2207-5

**Published:** 2017-06-26

**Authors:** Xianghai Ji, Xiaoguang Yang, Tao Yang

**Affiliations:** 1 Key Laboratory of Semiconductor Materials Science, Beijing Key Laboratory of Low Dimensional Semiconductor Materials and Devices, Institute of Semiconductors, Chinese Academy of Sciences, Beijing, 100083 People’s Republic of China; 2 College of Materials Science and Opto-Electronic Technology, University of Chinese Academy of Sciences, Beijing, 100049 People’s Republic of China

**Keywords:** Heterostructure nanowire, InAs/GaSb, Self-catalyzed, Crystal structure, Metal-organic chemical vapor deposition

## Abstract

We report the first self-catalyzed growth of high-quality GaSb nanowires on InAs stems using metal-organic chemical vapor deposition (MOCVD) on Si (111) substrates. To achieve the growth of vertical InAs/GaSb heterostructure nanowires, the two-step flow rates of the trimethylgallium (TMGa) and trimethylantimony (TMSb) are used. We first use relatively low TMGa and TMSb flow rates to preserve the Ga droplets on the thin InAs stems. Then, the flow rates of TMGa and TMSb are increased to enhance the axial growth rate. Because of the slower radial growth rate of GaSb at higher growth temperature, GaSb nanowires grown at 500 °C exhibit larger diameters than those grown at 520 °C. However, with respect to the axial growth, due to the Gibbs-Thomson effect and the reduction in the droplet supersaturation with increasing growth temperature, GaSb nanowires grown at 500 °C are longer than those grown at 520 °C. Detailed transmission electron microscopy (TEM) analyses reveal that the GaSb nanowires have a perfect zinc-blende (ZB) crystal structure. The growth method presented here may be suitable for other antimonide nanowire growth, and the axial InAs/GaSb heterostructure nanowires may have strong potential for use in the fabrication of novel nanowire-based devices and in the study of fundamental quantum physics.

**Electronic supplementary material:**

The online version of this article (doi:10.1186/s11671-017-2207-5) contains supplementary material, which is available to authorized users.

## Background

III–V semiconductor nanowires have been recognized as promising candidates for next-generation nanoscale electronic, optical, and quantum devices because of their unique electronic, optical, and geometrical properties [1–3]. Among the III–V semiconductor materials, because of their unique advantages, such as narrow direct bandgap, small carrier effective mass, and the highest carrier mobility, III-antimonides have strong potential for use in the fabrication of mid- and long-wave infrared photodetectors [4], low-power high-speed transistors [5–7] and in the study of fundamental quantum physics [8–10]. However, because of their heavy atomic mass, the low volatility of elemental Sb and the low melting temperature of III-antimonide compounds, achieving the growth of antimonide-based nanowires, is extremely challenging [11].

In particular, GaSb nanowires, considered to be extremely important p-type antimonide nanowires, have been mainly grown with the assistance of Au catalysts [12–16]. However, the introduction of Au may form unwanted deep-level recombination centers in the Si band gap and degrade the electronic and optical properties of III–V nanowires [17, 18]. Therefore, it is highly desirable to grow GaSb nanowires without any foreign catalysts. In addition, for vertical antimonide nanowire growth, direct nucleation on the substrate is very difficult. To avoid the nucleation issue, a short stem of another material is always grown first to assist the growth of vertical GaSb nanowires. Recently, the self-catalyzed growth of GaSb nanowires on GaAs stems has been realized by molecular beam epitaxy (MBE) [19], but to the best of our knowledge, there are no reports on the growth of high-quality GaSb nanowires without the use of foreign catalysts based on a metal-organic chemical vapor deposition (MOCVD) technique. Here, we present the self-catalyzed growth of GaSb nanowires with the assistance of InAs stems using MOCVD on Si (111) substrates. On the one hand, the growth of GaSb nanowires on InAs nanowire stems by a self-catalyzed mechanism is difficult due to the change of both anions and cations from the InAs stem to the upper GaSb. On the other hand, because of the low lattice mismatch of 0.6% and the unique type-II-broken band alignment between InAs and GaSb, the growth of GaSb nanowires on InAs stems to form InAs/GaSb axial heterostructure nanowires enables a new platform for many applications, including tunneling-based devices [7, 14, 20, 21], high-speed complementary metal-oxide-semiconductor (CMOS) transistors [22, 23], research on electron-hole hybridization [9], and exciton- and spin-physics studies [24].

In this article, high-quality GaSb nanowires with smooth sidewalls were achieved through careful control of the growth conditions. To achieve the growth of vertical InAs/GaSb heterostructure nanowires, relatively low flow rates of trimethylgallium (TMGa) and trimethylantimony (TMSb) were used first to preserve the Ga droplets on the InAs stems. Then, the TMGa and TMSb flow rates were increased to enhance the axial growth of the GaSb nanowires. Because of the slower radial growth rate of GaSb at higher growth temperature, GaSb nanowires grown at 500 °C have larger diameters than those grown at 520 °C. In addition, due to the Gibbs-Thomson effect and the reduction in the droplet supersaturation with increasing growth temperature, GaSb nanowires grown at 500 °C are larger in both diameter and length than those grown at 520 °C. Detailed transmission electron microscopy (TEM) analyses reveal that the crystal structure of the InAs stems is composed of a polytype of wurtzite (WZ) and zinc-blende (ZB) structures, while the axially grown GaSb nanowires have a pure ZB crystal structure completely free of planar defects.

## Methods

### Nanowire Growth

The InAs/GaSb heterostructure nanowires were grown by a close-coupled shower head MOCVD system (AIXTRON Ltd, Germany) at a chamber pressure of 133 mbar. Trimethylindium (TMIn) and TMGa were used as group III precursors, and arsine (AsH_3_) and TMSb were used as group V precursors. Ultra-high-purity hydrogen (H_2_) was used as a carrier gas, and the total flow rate of H_2_ was 12 slm. The nanowires were grown on Si (111) substrates. Prior to growth, the substrates were heated to 635 °C for annealing and then cooled to 400 °C under AsH_3_ flux to form (111)B-like surfaces [25]. The InAs stems were grown at 545 °C for 45 s with TMIn and AsH_3_ flow rates of 1.0 × 10^−6^ mol/min and 2.0 × 10^−4^ mol/min, respectively. Subsequently, the source fluxes were switched from TMIn and AsH_3_ to TMGa and TMSb, and the substrates were cooled to the specific temperature for the axial growth of GaSb nanowires. Finally, the samples were cooled to room temperature using TMSb as a protective agent.

### Characterization Methods

The morphology of the nanowires was characterized by scanning electron microscopy (SEM) (Nova Nano SEM 650), and TEM (JEM2010F TEM; 200 kV) in conjunction with X-ray energy dispersive spectroscopy (EDS) was used to investigate the crystal structure and the elemental composition distribution, respectively. For TEM observations, the as-grown nanowires were mechanically transferred from the samples to copper grids coated with a carbon film. Raman measurements were performed in backscattering geometry at room temperature using a 532-nm-wavelength laser as the excitation source (Jobin-Yvon HR Evolution Raman System). The samples were excited with a laser power of 0.36 mW over a spot-size of approximately 1 μm.

## Results and Discussion

**Fig. 1:**
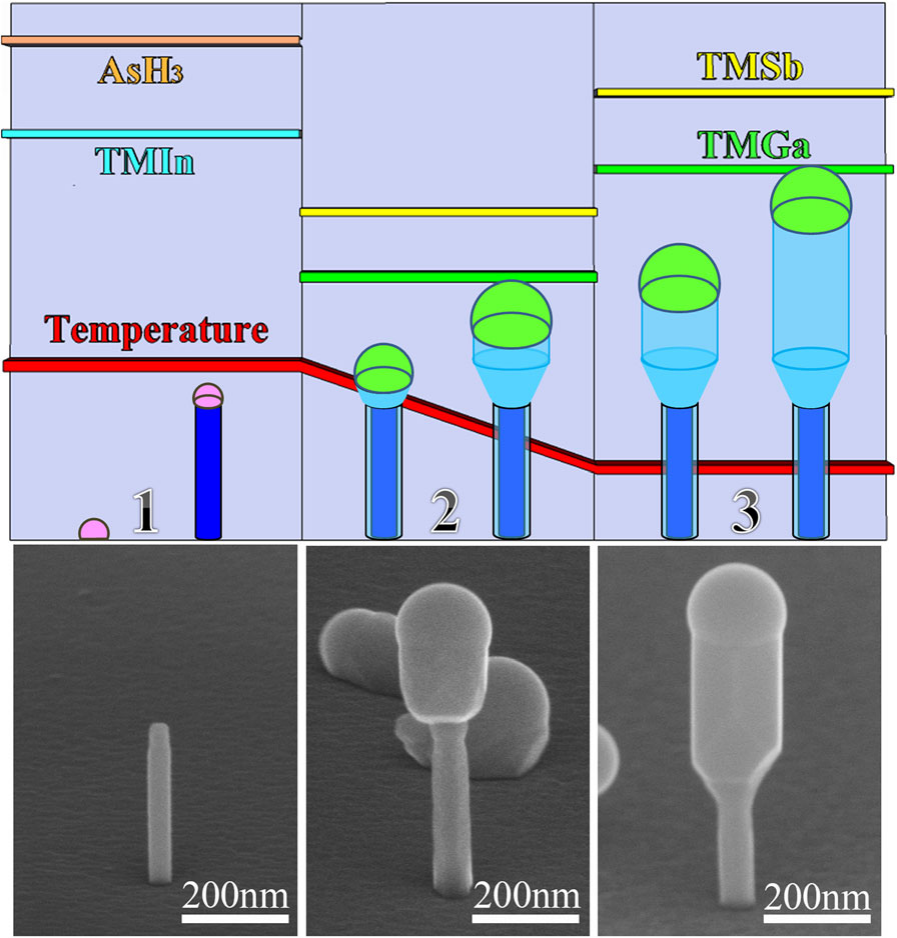
The illustration of the axial growth of GaSb nanowires on InAs stems and the source-supply sequences for the growth of the nanowires. The GaSb nanowires presented here were grown at 520 °C

Figure 1 shows a schematic illustration of the axial growth of GaSb nanowires on InAs stems and the source-supply sequences for the growth of the nanowires. The nanowires grow via a self-catalyzed mechanism, and catalytic droplets change from In to Ga gradually after switching the fluxes from TMIn and AsH_3_ to TMGa and TMSb. Compared to the stem nanowires, GaSb nanowires are always with a much thicker diameter, which means that the size of the Ga catalytic droplets is much larger than that of the In droplets. Then, overly rapid collection of Ga adatoms by the droplets on the thin InAs stems might cause a slipping down of the droplets (as shown in Additional file 1: Figure S1). To ensure that the catalytic droplets have enough time to collect Ga adatoms during the transition stage from InAs to GaSb, we first used relatively low flow rates of TMGa and TMSb to protect the Ga droplets on the InAs stems, as shown in Fig. 1. During the first step, the TMGa and TMSb flow rates were 0.35 × 10^−6^ mol/min and 2.0 × 10^−6^ mol/min, which correspond to a V/III ratio of ~5.7, and the growth process remained 15 min (region 2 in Fig. 1). After that, to increase the axial growth rate, the flow rates of TMGa and TMSb were increased to 0.7 × 10^−6^ mol/min and 4.0 × 10^−6^ mol/min for the subsequent growth of GaSb nanowires (keeping the V/III ratio constant), respectively. By using the two-step TMGa and TMSb flow rates, we successfully realized the vertical growth of GaSb nanowires on InAs stems. Considering the unchanged growth time of the GaSb nanowires with the low flow rates, unless specifically stated, the growth times for GaSb nanowires mentioned in the following paragraphs are identical to that of GaSb growth with the high flow rates (region 3 in Fig. 1).

**Fig. 2:**
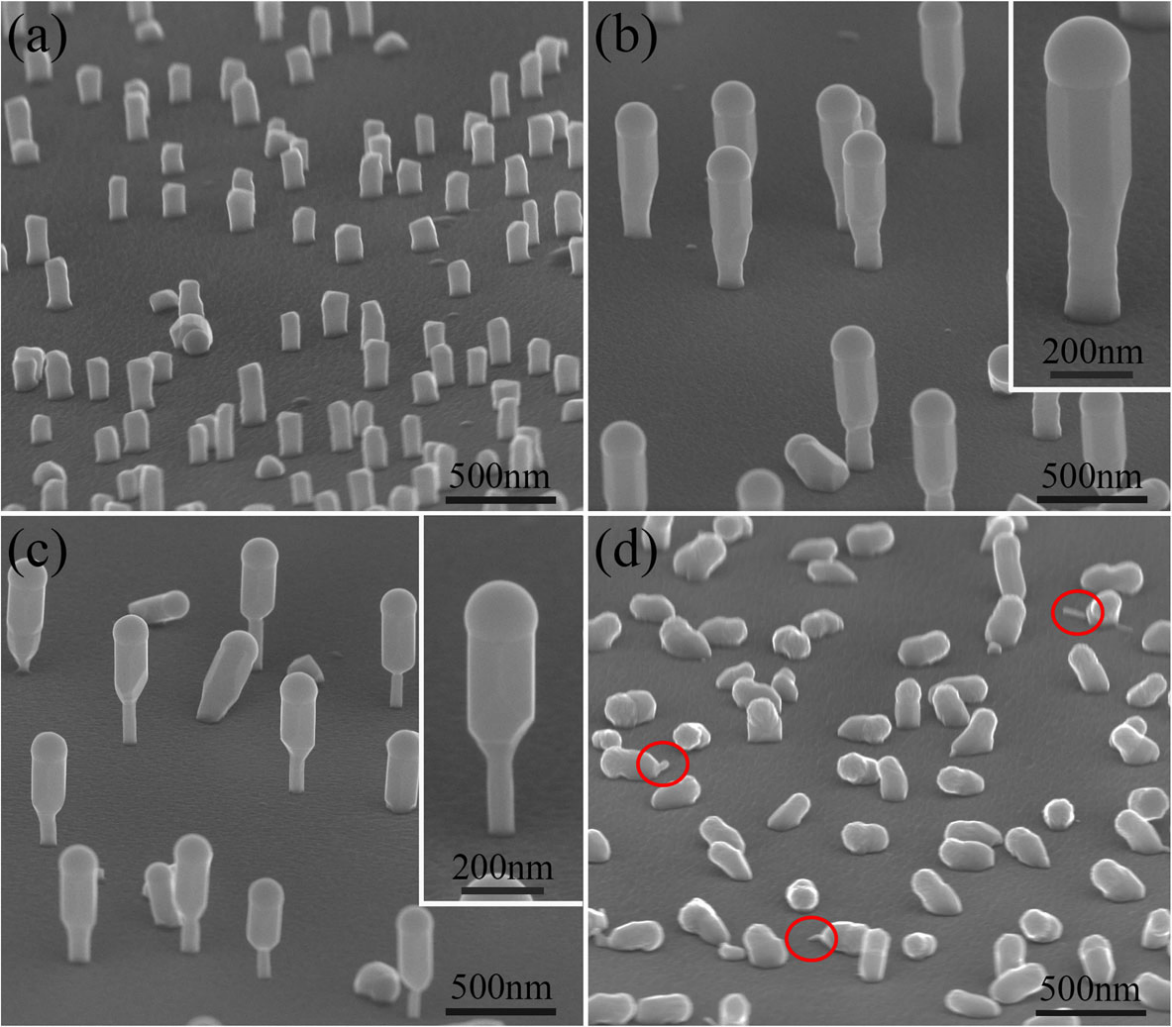
The 80°-tilted SEM images of the GaSb nanowires grown on InAs stems at **a** 480 °C, **b** 500 °C, **c** 520 °C, and **d** 545 °C for 20 min. The growth conditions of the InAs nanowire stems were kept constant. *Insets* in **b** and **c** show higher magnification SEM images. The *red circles* in **d** mark the residual InAs stems

Typical SEM images of the GaSb nanowires grown on InAs stems at different temperatures of 480, 500, 520, and 545 °C are shown in Fig. 2a–d, respectively (the InAs stems before the GaSb growth are shown in Additional file 1: Figure S2). The growth time of GaSb is 10 min. The growth behavior of the GaSb is observed to be very sensitive to the growth temperature. Clearly, at 480 °C, instead of axial growth, the GaSb tends to grow radially around the InAs stems or along the planar direction (Fig. 2a). Details about the radial growth of GaSb shells on InAs cores and the planar growth of antimonide nanowires have been reported elsewhere [26–28]. However, the situation changes as the GaSb growth temperature increases to 500 or 520 °C, where axial growth of GaSb nanowires is realized on the free-standing InAs stems (Fig. 2b, c). The Ga droplets at the nanowire tips indicate a self-catalyzed vapor-liquid-solid (VLS) growth mechanism of the GaSb nanowires. The diameter of the upper GaSb segments is generally thicker than that of the InAs segments, and the increasing width of the GaSb segments at the InAs/GaSb interface indicates that the size of the Ga catalytic droplets increases gradually at the initial growth stage of GaSb. In addition, the much thinner stem segments in Fig. 2c may imply that the radial growth of GaSb reduced gradually with the growth temperature increasing from 500 to 520 °C. However, when the temperature is further increased to 545 °C, GaSb nanowires seem like to be growing along the planar or the inclined direction. In addition, the most of InAs stems are disappeared and the diameter of the residual InAs stems is very thin (marked by the red circles in Fig. 2d, and more SEM images are shown in Additional file 1: Figure S3). We speculate that the InAs stems are seriously decomposed at the high growth temperature of 545 °C, resulting in the falling down of the GaSb nanowires during the growth process. Therefore, to obtain the vertical growth of GaSb nanowires on InAs nanowire stems, the GaSb nanowire growth temperature must be carefully controlled.

**Fig. 3:**
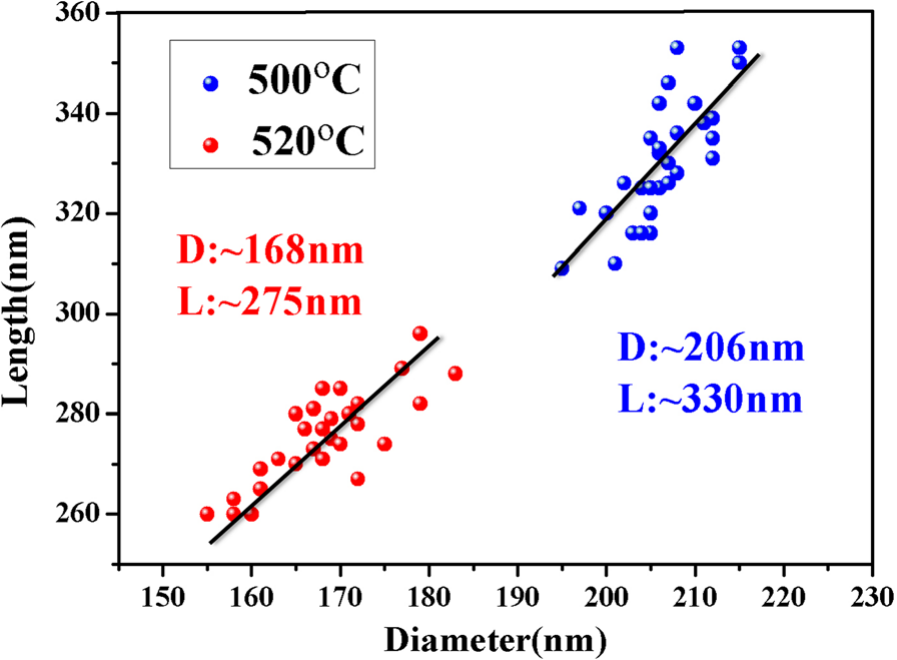
Statistical data of the diameter and length of the GaSb nanowires grown at 500 and 520 °C

Figure 3 displays the statistical distributions of the diameter and length of the GaSb segments shown in Fig. 2b, c, where the growth temperatures of GaSb are 500 and 520 °C, respectively. Clearly, the size distribution of GaSb nanowires grown at the same temperature (red or blue dots in Fig. 3) demonstrates that the thicker nanowires tend to be longer. This phenomenon has also been reported for the Ga-catalyzed growth of GaAsP nanowires by MBE [29] and for the Au-catalyzed growth of InAs/InSb heterostructure nanowires by chemical beam epitaxy (CBE) [30] and InGaSb nanowires by MOCVD [31]. The reason is mainly attributed to the lower effective supersaturation (Δ*μ*) in the smaller catalytic droplets. For the VLS growth mechanism, the supersaturation Δ*μ*, which is the change in chemical potential per III–V pair in the catalytic droplet and the nanowire, is the main driving force for the nanowire growth. During the self-catalyzed growth process, nanowires are grown in a group III-rich environment and the concentration of the group V atoms incorporated in the catalytic droplet dominates the effective supersaturation. For the self-catalyzed growth of GaSb nanowires, the effective supersaturation Δ*μ* is dominated by the concentration of Sb atoms incorporated in the Ga catalytic droplets. Therefore, the effective supersaturation Δ*μ* can be presented as [32, 33]

.

1 $$ \varDelta \mu ={k}_{\mathrm{B}} T \ln \left({x}_{\mathrm{Sb}}/{x}_{\mathrm{Sb},\mathrm{eq}}\right) $$

where *k*_B_ is the Boltzmann constant, *T* is the absolute temperature. *x*_Sb_ and *x*_Sb,eq_ are the atomic fraction of Sb in the Ga catalytic droplets during the nanowire growth process and at equilibrium with the GaSb nanowire crystal, respectively. Furthermore, based on the classic theory of crystal growth, the axial growth rate of the nanowire (*v*) can be expressed as [34].

2 $$ v\sim {\left(\varDelta \mu /{k}_{\mathrm{B}} T\right)}^2 $$

Clearly, the growth rate of nanowire has a strong dependence on the Sb concentration *x*_Sb_ in the Ga droplets. Because of the Gibbs-Thomson effect, the vapor pressure of Sb in the catalytic droplets can significantly increase as the diameter decreases [35, 36]. Then, the smaller droplets can more easily desorb Sb atoms from the Ga catalytic particles, which will result in the lower Sb concentration (*x*_Sb_) in the smaller Ga catalytic droplets. As a consequence, the effective supersaturation in the smaller droplets is lower than that in the larger ones, thereby reducing the axial growth rate of the GaSb nanowires by the self-catalyzed growth mechanism.

In addition, when comparing the size distributions of GaSb nanowires grown at 500 and 520 °C, the GaSb nanowires grown at 500 °C (blue dots in Fig. 3; average diameter and length ∼206 and ~330 nm) are observed to have both a larger diameter and length than the GaSb nanowires grown at 520 °C (red dots in Fig. 3; average diameter and length ∼168 and ~275 nm). The thinner nanowires grown at 520 °C can be attributed to the slower radial growth rate of GaSb at higher growth temperature. However, for the axial growth, other than the Gibbs-Thomson effect, the increase in the growth temperature can also reduce the droplet supersaturation and further reduce the axial growth rate of the GaSb nanowires [29, 37]. Thus, the GaSb nanowires grown at 500 °C are larger in both diameter and length than the GaSb nanowires grown at 520 °C.

**Fig. 4:**
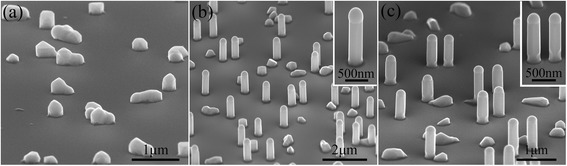
The 80°-tilted SEM images of the GaSb nanowires grown without InAs stems (**a**), and on the short InAs stems (**b**, **c**). The GaSb nanowires shown in **a** and **b** were grown at 500 °C, while the GaSb nanowires in **c** were grown at 520 °C. *Insets* in **b** and **c** show higher magnification SEM images

To further determine the benefits accruing from the assistance of the stem nanowires, we then compared the GaSb nanowires grown directly on Si substrates and on short InAs stems, as seen in Fig. 4. The GaSb nanowires shown in Fig. 4a, b were grown at 500 °C, while the GaSb nanowires presented in Fig. 4c were grown at 520 °C. Clearly, the InAs nanowire stems play a crucial role in the successful growth of vertical GaSb nanowires. As shown in Fig. 4a, the GaSb nanowires directly grown on Si (111) substrates prefer to grow along the planar direction (more SEM images are available in Additional file 1: Figure S4), and we speculate that this issue of antimonide nanowires directly nucleated on substrates is associated with the surfactant effect of Sb adatoms, which can decrease the contact angle between the pre-deposited Ga droplets and the Si substrate surface [38, 39]. Whereas, as shown in Fig. 4b, c, vertical GaSb nanowires are achieved with the assistance of the short InAs stems. We note that, in Fig. 4b, c, the growth time of the InAs nanowire stems is decreased to 20 s (resulting in the length of InAs nanowires being generally below 120 nm), while the growth time of the GaSb nanowires is increased to 30 min. All GaSb nanowires have extremely smooth sidewalls along their entire length without visible tapering. Notably, the InAs stem segments have almost the same diameter as the upper GaSb nanowires (as shown in the inset of Fig. 4b, c), which indicates that the radial growth rate of GaSb around InAs stems is faster than that around upper GaSb nanowires. This difference might be associated with the fact that the reactant adatoms diffused from the substrate surface tend to gather around the sidewalls of the thin and short InAs nanowires, resulting in the local enhancement of the radial growth rate of GaSb around the InAs stems. Finally, with enough growth time, the grown nanowires have an almost uniform diameter along the growth direction; the same behavior has also been observed in the growth of InSb nanowires based on short InAs stems by MOCVD [40].

**Fig. 5:**
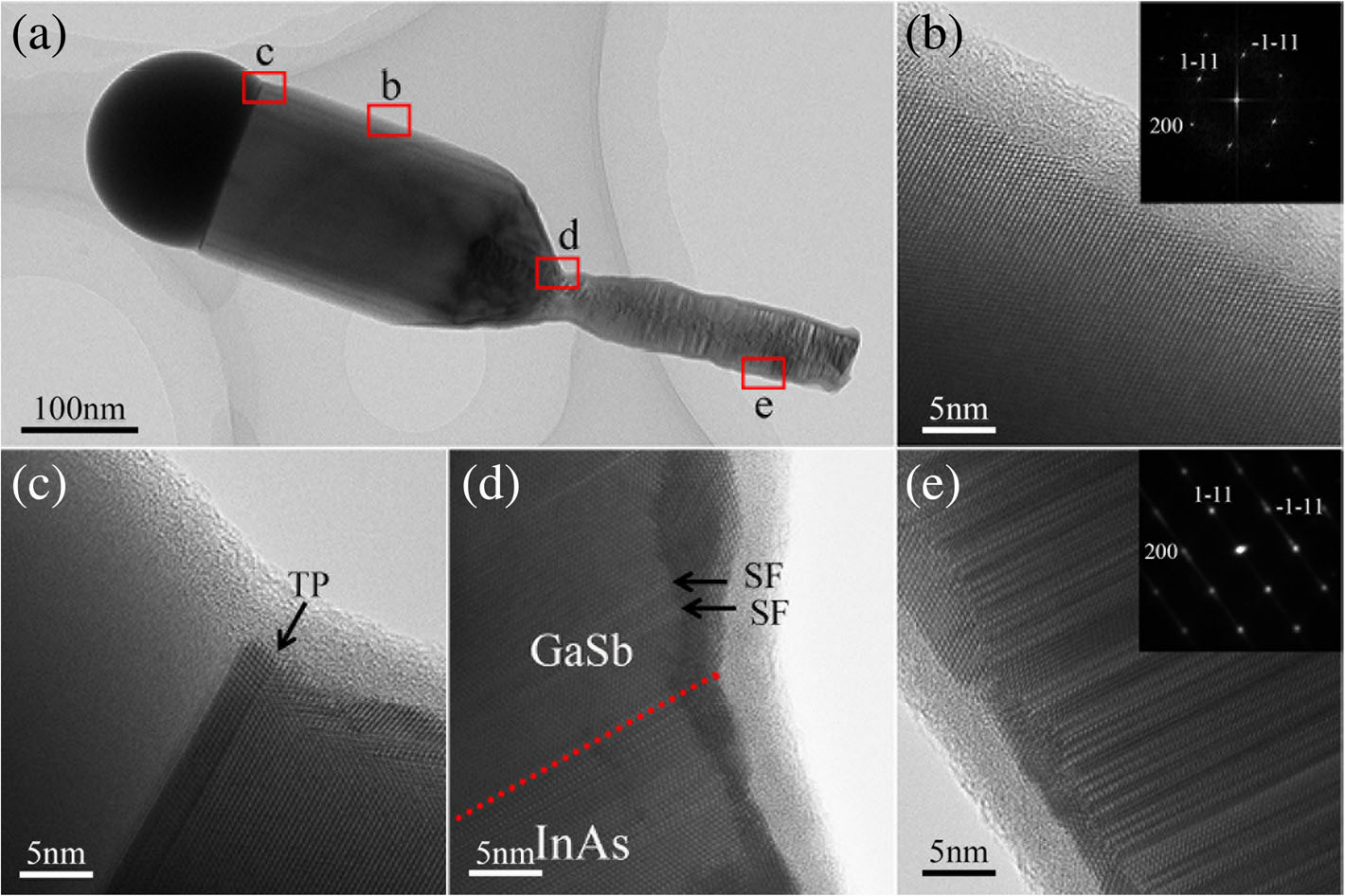
**a** A low-magnification TEM image of a typical InAs/GaSb heterostructure nanowire. **b**–**e** High-resolution TEM (HRTEM) images taken from the regions marked with *red rectangles* in (**a**) respectively. All HRTEM images are acquired from the 〈110〉 zone axis. The *red dashed line* in **d** indicates the interface between the InAs stem and the upper GaSb nanowire. The *insets* in **b** and **e** are the corresponding fast Fourier transform (FFT) patterns of the GaSb nanowire and the InAs stem, respectively

To examine the structural characteristics of the obtained nanowires, detailed TEM measurements were carried out. Figure 5a shows a bright-field (BF) low-resolution TEM image of a typical GaSb nanowire grown on an InAs stem at 520 °C (as shown in Fig. 2c). After the subsequent growth of the GaSb nanowire, the thinner InAs nanowire has a rough morphology. This could be attributed to the pyrolysis of InAs and the irregular radial growth of GaSb during the GaSb nanowire growth process. Figure 5b–e represent the corresponding high-resolution TEM (HRTEM) images taken from the regions marked with four red rectangles in Fig. 5a. The HRTEM micrograph and the associated fast Fourier transform (FFT) pattern in Fig. 5b reveal that the axially grown GaSb nanowire has a pure ZB crystal structure completely free of planar defects, which is commonly observed in the growth of antimonide nanowires. However, the occasional planar defects (twin planes (TPs) and stacking faults (SFs)) presenting at the top and early growth region of the GaSb nanowire (Fig. 5c, d) may be caused by a slight fluctuation of local growth conditions during the eventual cooling process and the initial transition stage from InAs to the upper GaSb. In addition, the residual As atoms can also play a role in the formation of planar defects in the transition region (as shown in the EDS analyses below). In contrast, as shown in Fig. 5e, the crystal structure of the InAs stem is composed of a polytype of WZ and ZB structures with a large number of planar defects along its growth direction; due to the coexistence of WZ and ZB structures, the corresponding FFT spots split and are slightly elongated along the growth direction (inset in Fig. 5e). Structural defects in nanowires have been shown to be able to cause undesirable inhibitions of carrier mobility [41] and hence reduce transport properties in the InAs/GaSb heterostructure system. Recently, the incorporation of Sb has been reported to effectively improve the crystal quality of InAs nanowires [42, 43], thereby greatly increasing the potential of the crystal-phase engineering of InAs nanowires without any foreign catalysts.

**Fig. 6:**
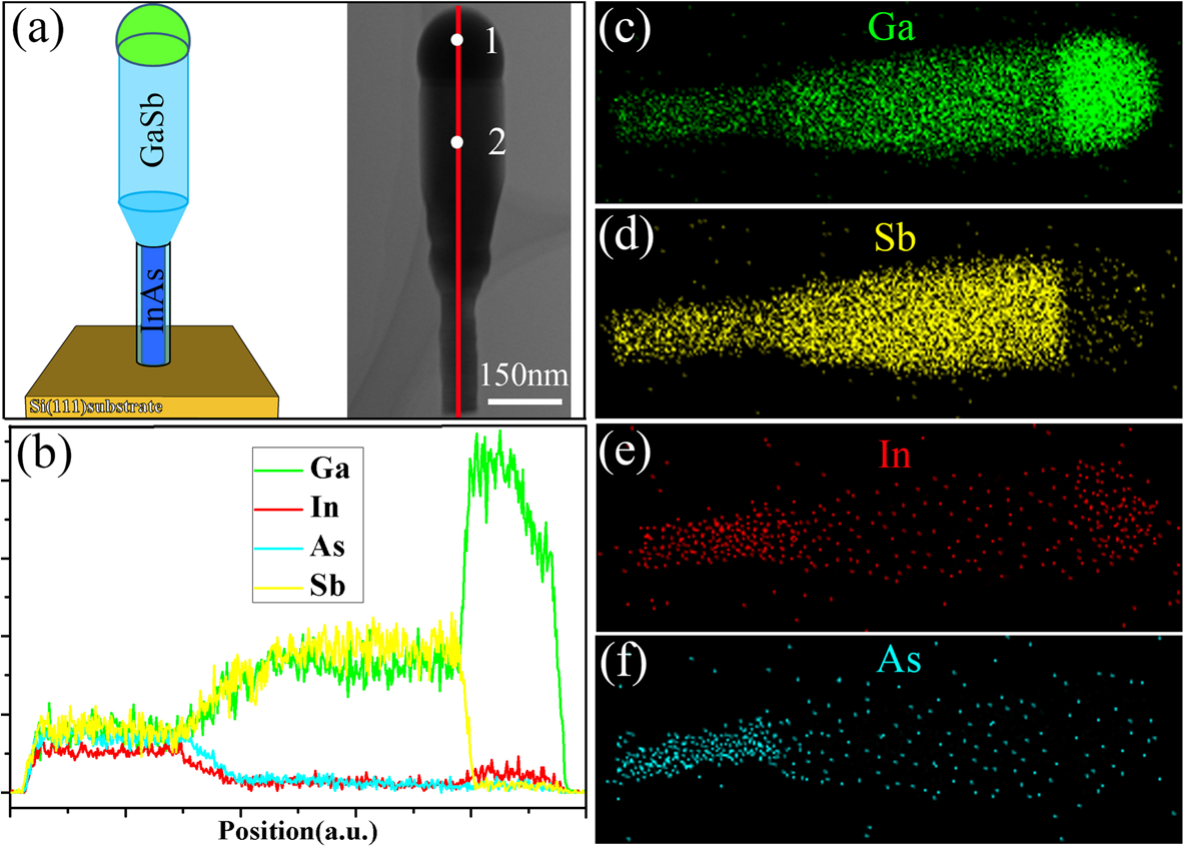
**a** The illustration of the axial InAs/GaSb heterostructure nanowires and a low-magnification TEM image of a grown InAs/GaSb nanowire grown at 520 °C. **b** EDS line scan along the *red line* marked in (**a**). **c**–**f** EDS compositional maps of the nanowire in (**a**), showing the Ga, Sb, In, and As distribution. *Two spots* in **a** mark the positions where EDS point analyses were performed

Figure 6a–f show a TEM image of another InAs/GaSb heterostructure nanowire and the corresponding EDS analyses. All EDS measurements use the Lα emission signals of In, As, and Sb and the Kα emission signal of Ga. The EDS line scan along the axial direction (Fig. 6b) and elemental mapping of the nanowire composition (Fig. 6c–f) show that the droplet mainly contains Ga and a small amount of In, while almost no As or Sb is observed, which directly confirms the self-catalyzed growth mechanism of the GaSb nanowires (atomic percentage from quantitative EDS point analysis in spot 1: Ga, 96.13%; In, 3.8%; As, 0; Sb, 0.07%, respectively. EDS spectra of point analyses in the two spots are shown in Additional file 1: Figure S5). This weak In concentration in the droplet is mainly attributed to the dissolution of the In atoms into the Ga droplet during the initial transition stage from InAs to GaSb. In addition, according to the EDS line scan in Fig. 6b, the growth of GaSb nanowires starts on the InAs stems, and from the EDS point analysis of the elemental composition distribution in spot 2 (Ga, 48.86%; In, 0.91%; As, 0.70%; Sb, 49.53%), the stoichiometric ratio of Ga and Sb atoms in the grown GaSb segment is approximately 1:1. However, the relatively high signals of Ga and Sb in the InAs section originate from the radial growth of the GaSb around the InAs stem, and the elemental gradient for the In, As, Ga, and Sb signals near the interface may be primarily caused by the residual In and As after the TMIn and AsH_3_ fluxes are shut off.

**Fig. 7:**
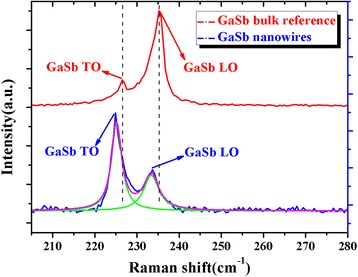
Raman spectra of a GaSb (100) substrate (*red line*) and the GaSb nanowires (*blue line*). The *green lines* are results of multi-peaks Lorentzian fit

To analyze the optical properties of the grown GaSb nanowires, Raman measurements were performed. Figure 7 shows the Raman spectra of a GaSb (100) substrate and the GaSb nanowires grown on the short InAs stems. Two scattering peaks are observed in the spectrum of the bulk GaSb at approximately 226.5 and 235.2 cm^−1^ (red line in Fig. 7) and are attributed to the transversal optical (TO) and longitudinal optical (LO) phonon modes of GaSb, respectively. For GaSb nanowires, similar two peaks were also observed clearly at approximately 225.0 and 233.6 cm^−1^ in the Raman spectrum (blue line in Fig. 7), indicating high photonics quality of the obtained GaSb nanowires. In Raman backscattering measurements, TO phonon mode is forbidden in the (100) direction, a small TO phonon mode peak for the bulk GaSb (100) substrate might be attributed to a slight substrate mis-orientation or imperfection [44]. However, for the GaSb nanowires, because the nanowires are grown along vertical (111) direction and with six {110} sidewalls, both the TO and LO phonon modes can be clearly observed in the Raman spectrum. In addition, compared with bulk GaSb, the TO and LO phonon modes of GaSb nanowires exhibit a weak downshift. In Raman scattering measurements, both the quantum confinement and defects can induce the frequency downshift of phonon peaks [45]. Whereas, because of the large diameter of the grown GaSb nanowires which shows almost no quantum confinement effect, we speculate that this weak downshift of phonon frequency may be associated with surface defects of the GaSb nanowires. With the two-step flow rates of the TMGa and TMSb, we have realized the vertical growth of pure ZB GaSb nanowires on InAs stems by MOCVD without any foreign catalysts. We expect that by further optimizing the growth parameters, such as the growth temperature and different combinations of the TMGa and TMSb flow rates in the two-step growth process, GaSb nanowires with a higher aspect ratio might be achieved.

## Conclusions

In summary, we have demonstrated the self-catalyzed growth of GaSb nanowires on InAs stems by MOCVD. To realize the growth of the vertical InAs/GaSb heterostructure nanowires, we first use relatively low TMGa and TMSb flow rates to preserve the Ga droplets on the InAs stems. Then, the flow rates of TMGa and TMSb are increased to improve the axial growth rate. Because of the slower radial growth rate of GaSb at higher growth temperature, GaSb nanowires grown at 500 °C have a larger diameter than those grown at 520 °C. However, for the axial growth, due to the Gibbs-Thomson effect and the reduction in the droplet supersaturation with the increasing growth temperature, the GaSb nanowires grown at 500 °C are longer than those grown at 520 °C. Detailed TEM measurements reveal that the crystal structure of the InAs stems is a mixture of WZ and ZB structures, while the upper GaSb nanowires have a perfect ZB crystal phase, and Raman analyses indicate high optical quality of the obtained GaSb nanowires. The growth method presented here may be suitable for the growth of other antimonide-based nanowires. Moreover, the as-grown GaSb nanowires on InAs stems may introduce new possibilities for applications in nanowire-based devices and for the study of quantum physics.

## Additional file

Additional file 1: A typical SEM image of the GaSb nanowires grown on InAs stems with relatively high TMGa and TMSb flow rates (S1), a typical SEM image of the InAs stems before the GaSb growth (S2), a typical SEM image of the GaSb nanowires grown on InAs stems at 545 °C (S3), a top-view SEM image of the GaSb nanowires directly grown on Si (111) substrates (S4), and EDS spectra of point analyses from different positions in a GaSb nanowire (S5). (DOCX 510 kb)

## Abbreviations

CMOS: Complementary metal-oxide-semiconductor
EDS: Energy dispersive spectroscopy
FFT: Fast Fourier transform
LO: Longitudinal optical
MBE: Molecular beam epitaxy
MOCVD: Metal-organic chemical vapor deposition
SEM: Scanning electron microscopy
SF: Stacking fault
TEM: Transmission electron microscopy
TMGa: Trimethylgallium
TMSb: Trimethylantimony
TO: Transversal optical
TP: Twin plane
VLS: Vapor-liquid-solid
ZB: Zinc-blende

## References

[1] Tomioka K, Yoshimura M, Fukui T (2012). A III-V nanowire channel on silicon for high-performance vertical transistors. Nature.

[2] Tatebayashi J, Kako S, Ho J, Ota Y, Iwamoto S, Arakawa Y (2015). Room-temperature lasing in a single nanowire with quantum dots. Nat Photonics.

[3] Cui Y, Wei QQ, Park HK, Lieber CM (2001). Nanowire nanosensors for highly sensitive and selective detection of biological and chemical species. Science.

[4] Svensson J, Anttu N, Vainorius N, Borg BM, Wernersson LE (2013). Diameter-dependent photocurrent in InAsSb nanowire infrared photodetectors. Nano Lett.

[5] Ganjipour B, Nilsson HA, Borg BM, Wernersson LE, Samuelson L, Xu HQ, Thelander C (2011). GaSb nanowire single-hole transistor. Appl Phys Lett.

[6] Thelander C, Caroff P, Plissard S, Dick KA (2012). Electrical properties of InAs1-xSbx and InSb nanowires grown by molecular beam epitaxy. Appl Phys Lett.

[7] Ganjipour B, Dey AW, Borg BM, Ek M, Pistol ME, Dick KA, Wernersson LE, Thelander C (2011). High current density Esaki tunnel diodes based on GaSb-InAsSb heterostructure nanowires. Nano Lett.

[8] Li S, Kang N, Fan DX, Wang LB, Huang YQ, Caroff P, Xu HQ (2016). Coherent charge transport in ballistic InSb nanowire Josephson junctions. Sci Rep.

[9] Ganjipour B, Leijnse M, Samuelson L, Xu HQ, Thelander C (2015). Transport studies of electron-hole and spin-orbit interaction in GaSb/InAsSb core-shell nanowire quantum dots. Phys Rev B.

[10] Mourik V, Zuo K, Frolov SM, Plissard SR, Bakkers EPAM, Kouwenhoven LP (2012). Signatures of Majorana Fermions in hybrid superconductor-semiconductor nanowire devices. Science.

[11] Mattias Borg B, Wernersson LE (2013). Synthesis and properties of antimonide nanowires. Nanotechnology.

[12] Gorji Ghalamestani S, Lehmann S, Dick KA (2016). Can antimonide-based nanowires form wurtzite crystal structure?. Nanoscale.

[13] Ek M, Borg BM, Johansson J, Dick KA (2013). Diameter limitation in growth of III-Sb-containing nanowire heterostructures. ACS Nano.

[14] Borg BM, Dick KA, Ganjipour B, Pistol ME, Wernersson LE, Thelander C (2010). InAs/GaSb heterostructure nanowires for tunnel field-effect transistors. Nano Lett.

[15] Guo YN, Zou J, Paladugu M, Wang H, Gao Q, Tan HH, Jagadish C (2006). Structural characteristics of GaSb∕GaAs nanowire heterostructures grown by metal-organic chemical vapor deposition. Appl Phys Lett.

[16] Jeppsson M, Dick KA, Wagner JB, Caroff P, Deppert K, Samuelson L, Wernersson L-E (2008). GaAs/GaSb nanowire heterostructures grown by MOVPE. J Cryst Growth.

[17] Breuer S, Pfuller C, Flissikowski T, Brandt O, Grahn HT, Geelhaar L, Riechert H (2011). Suitability of Au- and self-assisted GaAs nanowires for optoelectronic applications. Nano Lett.

[18] Allen JE, Hemesath ER, Perea DE, Lensch-Falk JL, Li ZY, Yin F, Gass MH, Wang P, Bleloch AL, Palmer RE, Lauhon LJ (2008). High-resolution detection of Au catalyst atoms in Si nanowires. Nat Nanotechnol.

[19] Yu X, Li L, Wang H, Xiao J, Shen C, Pan D, Zhao J (2016). Two-step fabrication of self-catalyzed Ga-based semiconductor nanowires on Si by molecular-beam epitaxy. Nanoscale.

[20] Rocci M, Rossella F, Gomes UP, Zannier V, Rossi F, Ercolani D, Sorba L, Beltram F, Roddaro S (2016). Tunable Esaki effect in catalyst-free InAs/GaSb core–shell nanowires. Nano Lett.

[21] Memišević E, Svensson J, Hellenbrand M, Lind E, Wernersson L-E (2016). Scaling of vertical InAs–GaSb nanowire tunneling field-effect transistors on Si. IEEE Electron Device Lett.

[22] Svensson J, Dey AW, Jacobsson D, Wernersson LE (2015). III-V nanowire complementary metal-oxide semiconductor transistors monolithically integrated on Si. Nano Lett.

[23] Dey AW, Svensson J, Borg BM, Ek M, Wernersson LE (2012). Single InAs/GaSb nanowire low-power CMOS inverter. Nano Lett.

[24] Abergel DSL (2015). Excitonic condensation in spatially separated one-dimensional systems. Appl Phys Lett.

[25] Tomioka K, Motohisa J, Hara S, Fukui T (2008). Control of InAs nanowire growth directions on Si. Nano Lett.

[26] Ji X, Yang X, Du W, Pan H, Luo S, Ji H, Xu HQ, Yang T (2016). InAs/GaSb core-shell nanowires grown on Si substrates by metal-organic chemical vapor deposition. Nanotechnology.

[27] Ji X, Yang X, Du W, Pan H, Yang T (2016). Selective-area MOCVD growth and carrier-transport-type control of InAs(Sb)/GaSb core–shell nanowires. Nano Lett.

[28] Du W, Yang X, Pan H, Ji X, Ji H, Luo S, Zhang X, Wang Z, Yang T (2016). Controlled-direction growth of planar InAsSb nanowires on Si substrates without foreign catalysts. Nano Lett.

[29] Zhang Y, Sanchez AM, Sun Y, Wu J, Aagesen M, Huo S, Kim D, Jurczak P, Xu X, Liu H (2016). Influence of droplet size on the growth of self-catalyzed ternary GaAsP nanowires. Nano Lett.

[30] Li A, Sibirev NV, Ercolani D, Dubrovskii VG, Sorba L (2013). Readsorption assisted growth of InAs/InSb heterostructured nanowire arrays. Cryst Growth Des.

[31] Ghalamestani SG, Ek M, Ganjipour B, Thelander C, Johansson J, Caroff P, Dick KA (2012). Demonstration of defect-free and composition tunable GaxIn1-xSb nanowires. Nano Lett.

[32] Joyce HJ, Wong-Leung J, Gao Q, Tan HH, Jagadish C (2010). Phase perfection in zinc Blende and Wurtzite III-V nanowires using basic growth parameters. Nano Lett.

[33] Zhou C, Zheng K, Lu Z, Zhang Z, Liao Z, Chen P, Lu W, Zou J (2015). Quality control of GaAs nanowire structures by limiting As flux in molecular beam epitaxy. J Phys Chem C.

[34] Givargizov EI (1975). Fundamental aspects of Vls growth. J Cryst Growth.

[35] Froberg LE, Seifert W, Johansson J (2007). Diameter-dependent growth rate of InAs nanowires. Phys Rev B.

[36] Dubrovskii VG, Sibirev NV, Cirlin GE, Soshnikov IP, Chen WH, Larde R, Cadel E, Pareige P, Xu T, Grandidier B, Nys JP, Stievenard D, Moewe M, Chuang LC, Chang-Hasnain C (2009). Gibbs-Thomson and diffusion-induced contributions to the growth rate of Si InP and GaAs nanowires. Phys Rev B.

[37] Glas F (2010). Chemical potentials for Au-assisted vapor-liquid-solid growth of III-V nanowires. J Appl Phys.

[38] Nebol'sin VA, Shchetinin AA (2003). Role of surface energy in the vapor-liquid-solid growth of silicon. Inorg Mater.

[39] Anyebe EA, Rajpalke MK, Veal TD, Jin CJ, Wang ZM, Zhuang QD (2015). Surfactant effect of antimony addition to the morphology of self-catalyzed InAs1 − x Sb x nanowires. Nano Res.

[40] Li TF, Gao LZ, Lei W, Guo LJ, Pan HY, Yang T, Chen YH, Wang ZG (2013). InAs-mediated growth of vertical InSb nanowires on Si substrates. Nanoscale Res Lett.

[41] Sourribes MJL, Isakov I, Panfilova M, Liu HY, Warburton PA (2014). Mobility enhancement by Sb-mediated minimisation of stacking fault density in InAs nanowires grown on silicon. Nano Lett.

[42] Du W-N, Yang X-G, Wang X-Y, Pan H-Y, Ji H-M, Luo S, Yang T, Wang Z-G (2014). The self-seeded growth of InAsSb nanowires on silicon by metal-organic vapor phase epitaxy. J Cryst Growth.

[43] Zhuang QD, Anyebe EA, Chen R, Liu H, Sanchez AM, Rajpalke MK, Veal TD, Wang ZM, Huang YZ, Sun HD (2015). Sb-induced phase control of InAsSb nanowires grown by molecular beam epitaxy. Nano Lett.

[44] Su YK, Gan KJ, Hwang JS, Tyan SL (1990). Raman spectra of Si-implanted GaSb. J Appl Phys.

[45] Li T, Chen Y, Lei W, Zhou X, Luo S, Hu Y, Wang L, Yang T, Wang Z (2011). Effect of growth temperature on the morphology and phonon properties of InAs nanowires on Si substrates. Nanoscale Res Lett.

