# Supplementary material for: Self-Catalyzed Growth of Vertical GaSb Nanowires on InAs Stems by Metal-Organic Chemical Vapor Deposition
Source: Nanoscale Res Lett. 2017 Jun 26;12:428. doi: 10.1186/s11671-017-2207-5 (PMC5484658; doi:10.1186/s11671-017-2207-5)
Supplement: Additional file 1: — A typical SEM image of the GaSb nanowires grown on InAs stems with relatively high TMGa and TMSb flow rates (S1), a typical SEM image of the InAs stems before the GaSb growth (S2), a typical SEM image of the GaSb nanowires grown on InAs stems at 545 °C (S3), a top-view SEM image of the GaSb nanowires directly grown on Si (111) substrates (S4), and EDS spectra of point analyses from different positions in a GaSb nanowire (S5). (DOCX 510 kb) [file 11671_2017_2207_MOESM1_ESM.docx]

**Additional file**

**Self-catalyzed growth of vertical GaSb nanowires on InAs stems by metal-organic chemical vapor deposition**

Xianghai Ji,^1,2^ Xiaoguang Yang, ^1,2^ and Tao Yang ^1,2^

^1^ Key Laboratory of Semiconductor Materials Science, Beijing Key Laboratory of Low Dimensional Semiconductor Materials and Devices, Institute of Semiconductors, Chinese Academy of Sciences, Beijing 100083, People’s Republic of China

^2^ College of Materials Science and Opto-Electronic Technology, University of Chinese Academy of Sciences, Beijing 100049, People’s Republic of China

***E-mail:** [**tyang@semi.ac.cn**](mailto:tyang@semi.ac.cn)

**S1 A typical SEM image of the GaSb nanowires grown on InAs stems with relatively high TMGa and TMSb flow-rates.**

Figure S1 shows a typical SEM image of the GaSb nanowires grown on InAs stems at 520 °C for 20 min. The flow-rates of TMGa and TMSb are 0.7×10^-6^ mol/min and 4.0×10^-6^ mol/min, respectively. However, instead of vertically growing on the InAs stems, GaSb nanowires are observed to grow along the planar direction on the substrate surface. Moreover, their tails of many GaSb nanowires are sticking to InAs stems, which implies that the initial nucleation of these GaSb nanowires starts on the side facet of the InAs stems. Generally, compared to the InAs stems, the upper GaSb nanowires always have a much thicker diameter, which means that the size of the Ga catalytic droplets is much larger than that of the In droplets. We speculate that overly rapid-collection of Ga adatoms by the droplets on the thin InAs stems might cause a slipping-down of the droplets, resulting in the planar growth of GaSb nanowires from the InAs stems. Therefore, to realize the axial growth of GaSb nanowires on InAs stems, the flow rates of precursors need to be controlled carefully.


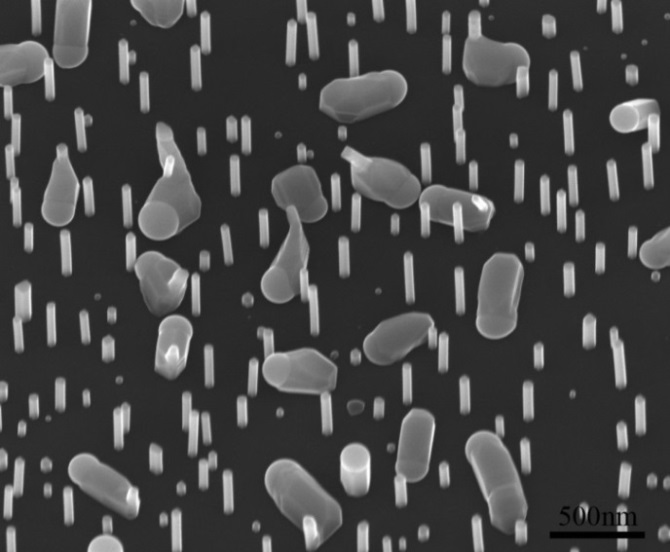


**Figure S1** A typical 45°-tilted SEM image of the GaSb nanowires grown on InAs stems with TMGa and TMSb flow-rates of 0.7×10^-6^ mol/min and 4.0×10^-6^ mol/min at 520 °C for 20 min.

**S2 A typical SEM image of the InAs stems before the GaSb growth.**

Figure S2 shows a SEM image of the InAs nanowires before the GaSb growth. The nanowires were observed to be vertically grown on the Si (111) substrate with smooth sidewalls. Compared with the InAs stems, the nanowires shown in Fig. 2a in the article are with thicker diameter and quite rough morphology, which confirm that the GaSb grows radially around the InAs stems in Fig. 2a in the article.


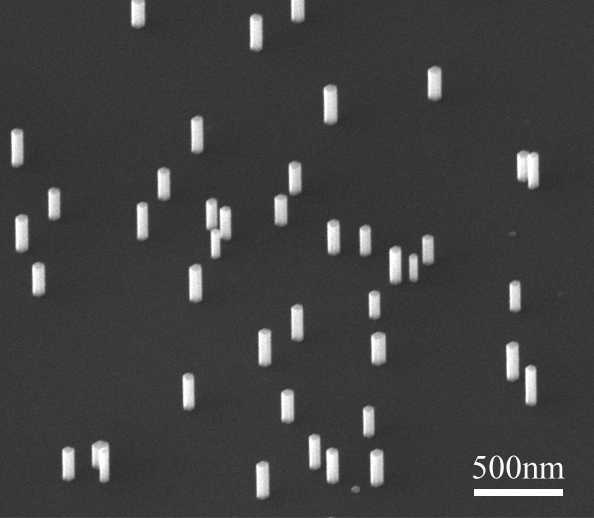


**Figure S2** A typical 45°-tilted SEM image of the InAs nanowires before the GaSb growth. The growth time was 45 s.

**S3 A typical SEM image of the GaSb nanowires grown on InAs stems at 545 °C.**

Figure S3 shows a typical 80°-tilted SEM image of the GaSb nanowires grown on InAs stems at 545 °C for 20 min. The InAs stems can be occasionally observed. Compared with the InAs stems before the GaSb growth, the diameter of residual InAs stems are much thinner. Therefore, we speculate that the InAs stems are seriously decomposed at the high growth temperature of 545 °C (most of the InAs stems are decomposed completely), resulting in the falling-down of the GaSb nanowires during the growth process.


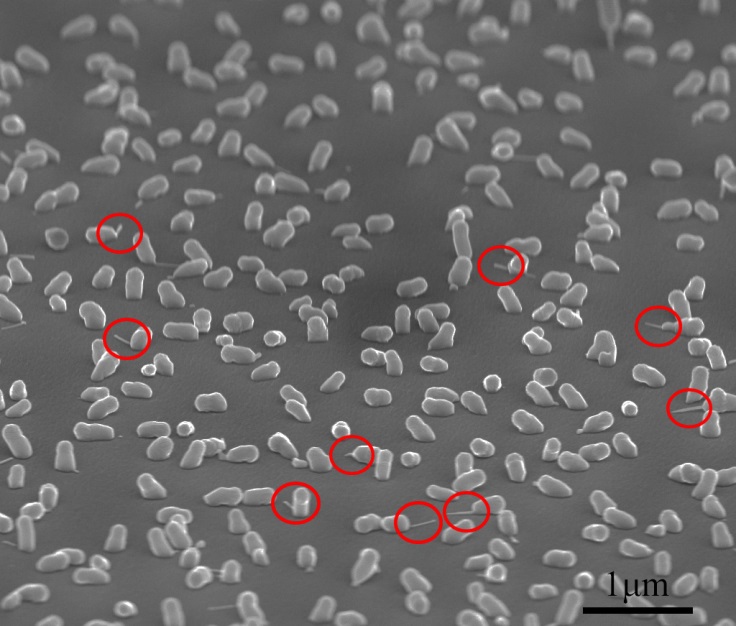


**Figure S3** A typical 80°-tilted SEM image of the GaSb nanowires grown on InAs stems at 545 °C for 20 min. The red circles mark some of the residual InAs stems.

**S4** **A top-view SEM image of the GaSb nanowires directly grown on Si (111) substrates.**

Figure S4 shows a top-view SEM image of the GaSb nanowires directly grown on Si (111) substrates at 500 °C. Clearly, the GaSb nanowires grown on bare Si (111) substrates prefer to grow along the planar direction, and the growth occurred along six specific in-plane directions which correspond to the projections of the six ⟨111⟩ family crystal directions on the (111)-oriented substrate surface (as shown in the inset in Figure S4, the angles between each direction are 60°).


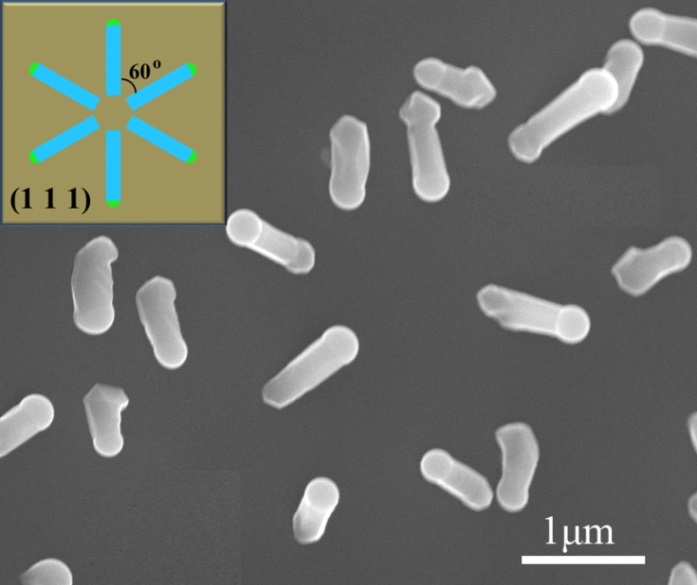


**Figure S4** A top-view SEM image of the GaSb nanowires directly grown on Si (111) substrates.

**S5 EDS spectra of point analyses from different positions in a GaSb nanowire.**


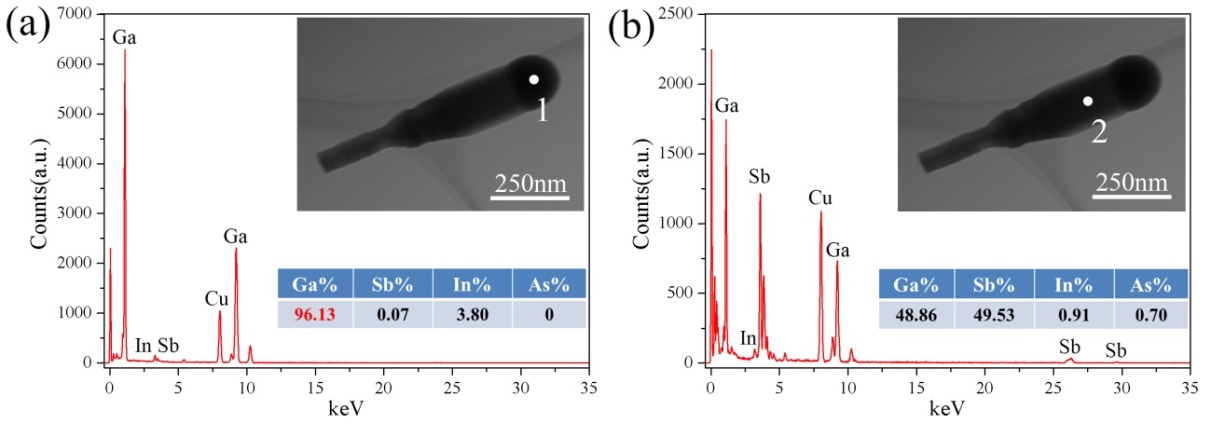


**Figure S5** (a), (b) EDS spectra of point analyses from the different spots (position 1, 2) in the GaSb nanowire, respectively. Atomic percentages from quantitative EDS point analyses in two spots are of (a) Ga, 96.13%; Sb, 0.07%; In, 3.8%; As, 0 and (b) Ga, 48.86%; Sb, 49.53%; In, 0.91%; As, 0.70%, respectively.
